# Selection of relatively exact reference genes for gene expression studies in goosegrass (*Eleusine indica*) under herbicide stress

**DOI:** 10.1038/srep46494

**Published:** 2017-04-21

**Authors:** Jingchao Chen, Zhaofeng Huang, Hongjuan Huang, Shouhui Wei, Yan Liu, Cuilan Jiang, Jie Zhang, Chaoxian Zhang

**Affiliations:** 1 Key Laboratory of Weed and Rodent Biology and Management, Institute of Plant Protection, Chinese Academy of Agricultural Sciences, Beijing 100193, P. R. China; 2 Environment and Plant Protection Institute, Chinese Academy of Tropical Agricultural Sciences, Danzhou 571737, P. R. China; 3 State Key Laboratory for Biology of Plant Diseases and Insect Pests, Institute of Plant Protection, Chinese Academy of Agricultural Sciences, Beijing 100193, P. R. China

## Abstract

Goosegrass (*Eleusine indica*) is one of the most serious annual grassy weeds worldwide, and its evolved herbicide-resistant populations are more difficult to control. Quantitative real-time PCR (qPCR) is a common technique for investigating the resistance mechanism; however, there is as yet no report on the systematic selection of stable reference genes for goosegrass. This study proposed to test the expression stability of 9 candidate reference genes in goosegrass in different tissues and developmental stages and under stress from three types of herbicide. The results show that for different developmental stages and organs (control), eukaryotic initiation factor 4 A (*eIF-4*) is the most stable reference gene. Chloroplast acetolactate synthase (*ALS*) is the most stable reference gene under glyphosate stress. Under glufosinate stress, *eIF-4* is the best reference gene. Ubiquitin-conjugating enzyme (*UCE*) is the most stable reference gene under quizalofop-p-ethyl stress. The gene *eIF-4* is the recommended reference gene for goosegrass under the stress of all three herbicides. Moreover, pairwise analysis showed that seven reference genes were sufficient to normalize the gene expression data under three herbicides treatment. This study provides a list of reliable reference genes for transcript normalization in goosegrass, which will facilitate resistance mechanism studies in this weed species.

Weeds are one of the major biotic threats to crops and can result in a 34% loss of crop yield worldwide^1^. Herbicides are a class of chemicals that can control weeds efficiently and economically, enhance crop yield and liberate the workforce. Unfortunately, over-reliance on herbicides has resulted in the evolution of resistance in weed species^2^. To date, 251 weed species in 66 countries have been found to have evolved resistance to 160 different herbicides^3^. Goosegrass (*Eleusine indica*) is considered one of the top ten worst weeds, distributed worldwide and affecting crops^4^. Acetyl-CoA carboxylase (ACCase)-inhibiting herbicides and non-selective herbicides such as glyphosate, glufosinate and paraquat are widely used to control the goosegrass^5^. After many years of control by herbicides, goosegrass has evolved resistance to many of them, including glyphosate^6^,^7^, diclofop^8^, fluazifop^9^, glufosinate^10^ and paraquat^11^,^12^.

Clarifying the resistance mechanism for weeds is essential to control them, ensuring crop yield and the sustainable application of herbicides^13^. In general, resistance mechanisms can be categorized as target and non-target^14^. Gene expression analysis is commonly applied to explore target-site gene amplification and the over-expression of detoxifying enzyme genes, which are related to herbicide resistance^15^,^16^,^17^. Quantitative real-time PCR (qPCR) is necessarily in wide use^18^. Normalization during qPCR analysis is usually performed using a reference gene that must be expressed at stable levels regardless of the experimental conditions to ensure the veracity of the qPCR analysis^19^,^20^. Several studies have been published with the aim of identifying suitable reference genes for expression analysis under different conditions in plants, including weeds^21^,^22^,^23^,^24^. However, few studies focus on the stress under herbicide treatment for weeds^25^,^26^,^27^,^28^. With the development of next-generation sequencing technologies that can be used to investigate resistance mechanisms for weeds, the expression levels of increasing numbers of genes should be validated. Thus, the selection of a stably expressed reference gene is important for the accuracy of the results.

Studies of the resistance mechanism of goosegrass to herbicides have been conducted at both the phenotypic character and the molecular levels^7^,^29^,^30^. Acetyl-CoA carboxylase (*ACCase*), 5-enolpyruvylshikimate-3 phosphate synthase (*EPSPS*) and glutamine synthetase (*GS*) are three common genes whose expression levels are affected in resistant goosegrass and constitute the target-set of quizalofop-p-ethyl glyphosate and glufosinate^8^,^15^. However, no systematic analysis of reference gene selection for normalization in various development stages of goosegrass and the herbicide stress has previously been performed. In this study, 9 selected reference genes were cloned and identified in goosegrass, and then the stability of gene expression was assessed in different developmental stages, different organs, and different times after treatment with three herbicides. The aim of this study is to provide useful guidelines for future gene expression studies in goosegrass.

## Results

### RNA isolation and primer selection

**Figure 1:**
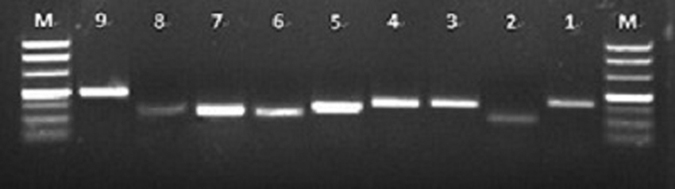
Amplification products of 9 candidate reference genes from *E. indica* by normal PCR. M: DL 500 maker. Lanes 1, 2, 3, 4, 5, 6, 7, 8 and 9 were the genes *ACTIN, GAPDH, ALS, EF, eIF-4, TUA, TUB, UCE* and *HSP* from *E. indica*, respectively.

The RNA quality of all samples was detected by both 1% agarose gel electrophoresis and A260/A280 values. All selected samples in this study showed a specificity band, and the values of A260/A280 ranged from 1.90 to 2.11 (Supplementary Figure S1). The 9 candidate reference genes in this study were retrieved from the transcriptome database of *E. indica*. Partial sequences of the 9 reference genes were reconfirmed by the PCR results and submitted to the NCBI database (Table 1). At least three primer pairs were designed, and the selected primers showed amplification specificity by gel electrophoresis. Then, the candidate primer pairs were detected by qPCR based on a standard curve. The results showed that all the appropriate primers exhibited a single band with the expected size by gel electrophoresis (Fig. 1) and a single peak in the melting curve (Supplementary Figure S2). In addition, all the primer pairs showed acceptable amplification efficiency (E), which ranged from 90.12% to 97.25%, and the correlation coefficients (R^2^) varied from 0.990 to 0.998 (Table 1; Supplementary Figure S3).

### Expression profiles of candidate reference genes

**Figure 2:**
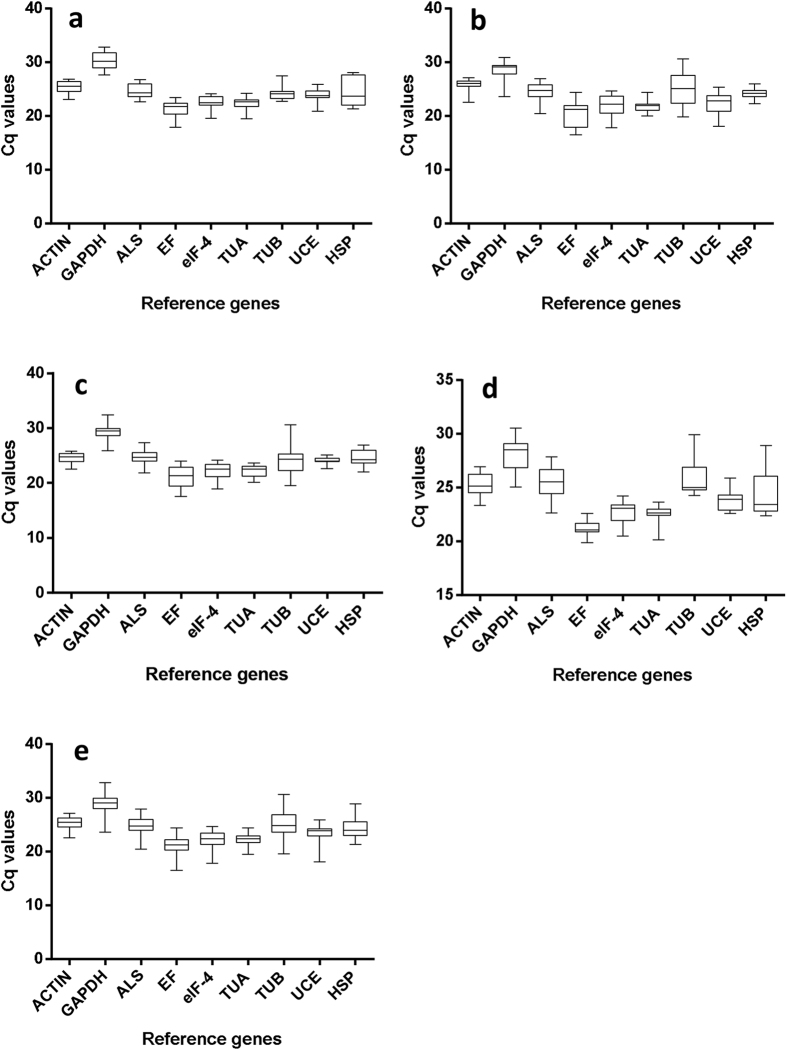
Expression levels of reference genes tested under different experimental conditions. (**a**) Control (different developmental stage and organs); (**b**) under glyphosate stress; (**c**) under glufosinate stress; (**d**) under quizalofop-p-ethyl stress; and (**e**) total.

The expression levels of the 9 candidate reference genes in goosegrass under different conditions were determined by the ABI7500, and the results were shown as quantification cycles^31^. According to the box-plot, the Cq values of the 9 reference genes displays a diversity ranging from 16.52 to 32.86, and their expression patterns are similar under different treatment (Fig. 2). The gene elongation factor 1-alpha (*EF*) showed the low Cq values ranging from 16.52 to 24.37, indicating high expression. In contrast, the gene glyceraldehyde-3-phosphate dehydrogenase (*GAPDH*) displayed high Cq values ranging from 23.57 to 32.86, corresponding to low expression. Compared across the different developmental stages and organs (control), the expression of the gene beta-tubulin 4 (*TUB*) was affected significantly under herbicide stress. The standard deviation (SD) of the samples for the control was 1.42, while the SD value can reach 3.55 under herbicide treatment.

### GeNorm analysis

This software evaluated the stability level of the reference genes by two parameters, stability value (M) and pairwise variation (V_n_/V_n+1_). The lowest M value means the most stable gene expression. The reference gene was accepted if the M value was below 1.5. In our research, *eIF-4* and *EF* ranked as the most stable genes under the control experiment set. Alpha tubulin 1 (*TUA*) is considered the least stable gene, with an M value of 1.48, and two genes (*TUB* and heat shock protein 70 (*HSP*) were not accepted by geNorm because the M values were greater than 1.5 (Table 2; Supplementary Figure S4). Under the stress of glyphosate, glufosinate and the total set, the most stable genes were the same (eukaryotic initiation factor 4A (*eIF-4*) and chloroplast acetolactate synthase (*ALS*), while *eIF-4* and ubiquitin-conjugating enzyme (*UCE*) were ranked as the most stable genes under quizalofop-p-ethyl stress. In addition, to define the best pair using geNorm, we also estimated the pairwise variation (V_n_/V_n+1_) to determine the minimal number of genes for reliable normalization with a cut-off value of 0.15. The pairwise variation was more than 0.15 in all the experiment sets (Supplementary Figure S5). As recommended in this case^32^, we used the lowest V_n/n+1_ value to determine the number of reference genes adequate for normalization. Under the stress of the three types of herbicide, using the most seven stable reference genes is considered to be a valid normalization strategy. Under the control and total conditions, the eight most stable reference genes should be used.

### NormFinder analysis

The stability value is the parameter used to evaluate the appropriate reference genes by NormFinder, where the lower stability value indicates higher expression stability^20^. In this research, the results analysed by NormFinder were similar to the analysis by geNorm. Under glufosinate stress, quizalofop-p-ethyl stress and for all samples, the most and least stable reference genes were the same as in the results evaluated by the geNorm, i.e., *eIF-4* and *TUB*, respectively (Table 2). The gene *eIF-4* was ranked as the most stable (0.24) under the control experiment set, while *HSP* was the most varied gene (1.33). *ALS* was the most stable gene (0.71) under glyphosate stress, while the least stable gene was *TUB* (1.79).

### BestKeeper analysis

The algorithm of the BestKeeper software is different from geNorm and NormFinder, depending on the SD and coefficient of variance (CV). The lowest SD and CV indicate the most stable reference gene. In addition, the parameter SD for the accepted reference gene must be lower than 1.0. The results analysis by BestKeeper differed from the results analysis by geNorm and NormFinder, with no more than five accepted reference genes in the entire experimental set (Table 2). Under the control set, total samples and quizalofop-p-ethyl stress conditions, the *TUA* was ranked as the most stable reference gene, while the most stable genes under glyphosate and glufosinate stress were *HSP* and *UCE*, respectively. The gene *ACTIN* was acceptable under all the experimental set.

### ReFinder analysis

To address the heterogeneity of the results evaluated by the geNorm, NormFinder and BestKeeper, another web tool ReFinder was used, which can compare the data generated by the other three software programs to give a comprehensive ranking confirming the stability ranking via different programs. The results show that the most stable reference genes were *eIF-4* (1.41), *ALS* (1.50), *eIF-4* (1.63), *UCE* (1.32) and *eIF-4* (1.50) for the control, glyphosate, glufosinate, quizalofop-p-ethyl and total set (Table 2).

### Expression profiles of *EPSPS, GS* and *ACCase*

**Figure 3:**
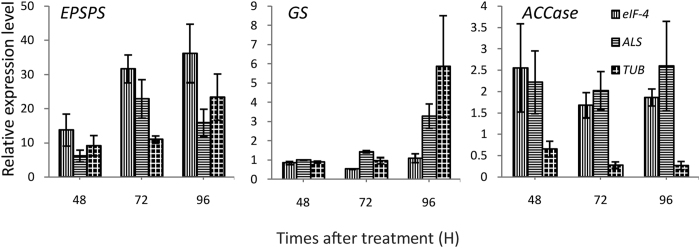
Relative normalized expression of *EPSPS, GS* and *ACCase* in leaf tissue of *E. indica* under glyphosate, quizalofop-p-ethyl, and glufosinate treatment, respectively.

The expression of the target-site genes of glyphosate (*EPSPS*), glufosinate (*GS*) and quizalofop-p-ethyl (*ACCase*) were determined in the leaf tissue after treatment at different times. Both the most stable reference genes (*eIF-4, ALS*) and the most varying reference gene (*TUB*) were chosen for this study. *ALS* is the most stable reference gene for goosegrass under glyphosate stress. Using *ALS* as the reference gene, the expression of *EPSPS* at 72 h and 96 h is 3.7 and 2.8 times higher than at 48 h after glyphosate treatment, respectively. When using the *TUB*, it was only 1.2 times and 0.7 times, respectively (Fig. 3). For *GS*, the expression at 96 h is 1.3 times higher than at 48 h using the best reference gene *eIF-4*. The most variable reference gene, *TUB*, can produce a result of up to 5.6 times. The expression of *ACCase* in goosegrass after quizalofop-p-ethyl treatment was altered only slightly. However, a difference can still be found between the more and less stable reference genes. The expression at 48 h is 1.5 times the expression at 72 h when using *eIF-4*, while the result is 2.4 using the least stable gene, *TUB*.

## Discussion

Herbicide-resistant weeds are a significant problem for crop production worldwide. Countering the resistance mechanism can provide a beneficial way to control them and ensure food safety. The qPCR method is the most common way to determine the expression profiles for genes related to herbicide resistance in weeds^33^,^34^, especially with the application of the technology of next-generation sequencing to investigate the resistance mechanism^15^,^35^,^36^. The selection of a stable reference gene is crucial for the accuracy of the qPCR results. However, little research has focused on finding reference gene for weeds, including goosegrass, under herbicide stress^27^,^28^. In this research, we selected stable reference genes from 9 candidate reference genes under the stress of three herbicides. The results showed that the most stable reference gene is *ALS* under glyphosate stress, *eIF-4* under glufosinate stress, and *UCE* under quizalofop-p-ethyl stress. The gene *eIF-4* is the best candidate reference gene in goosegrass under stress from all three herbicides.

The first report to identify reference genes under the herbicide stress of *ACCase* inhibitor is on *Alopecurus myosuroides*, and ubiquitin (*UBQ*), *TUB*, and *GAPDH* were selected for normalization^27^. In our research, the most stable gene under the ACCase inhibitor quizalofop-p-ethyl was *UCE*, followed by *eIF-4* and *EF*, while *TUB* was the least stable gene for goosegrass. Another study found that the most stable genes for *Avena fatuaunder* the stress of ACCase and ALS inhibitor herbicides were TATA-binding protein (*TBP*) and *GAPDH*^26^. Compared to this research, the most stable reference genes under the stress of the *ALS* inhibitor herbicide tribenuron were *ACT7* and *UBC* for *Descurainia sophia*^25^. These results indicated that the selection of the stable gene differed for different species. Herbicide stress is a type of abiotic stress, and the gene *eIF-4* showed stable expression in our research. This result was similar to other findings in grasses under different stresses^37^,^38^,^39^,^40^. Glyphosate is a very important herbicide due to the development of transgenic technology. Unfortunately, 36 weed species have evolved resistance to glyphosate thus far^3^. Many studies focus on investigating the resistance mechanism using qPCR; however, fewer studies systematically select the reference gene^34^,^41^,^42^. In our research, *ALS* is the most stable reference in *E. indica* under glyphosate stress, followed by *TUA* and *eIF-4*. The same results were found for *Amaranthus palmeri* and *Kochia scoparia*, where *ALS* was selected as the reference gene^33^,^42^. In our previous study, *ACTIN* was found to be more stable than *GAPDH*, as calculated by the software Bestkeeper^16^. This finding is consistent with the results in this study. However, the results of the stability ranking calculated by the software geNorm and NormFinder were different from the results calculated by Bestkeeper. This heterogeneity was also found in other plant species and may be due to the different algorithms for the software geNorm and NormFinder^24^. Fortunate, the software RefFinder can generate a comprehensive ranking of reference genes for all the other software.

Many research also focus on the genes expression in different tissues and developmental stages. We selected the stable expression reference genes in goosegrass under three different tissues and growing stages, respectively. Both the geNorm and NormFinder calculated the *eIF-4* as the most stabile reference. While, *TUA* was ranking as the most stabile gene used the Bestkeeper. All the three softwares ranked *HSP* as the least stabile reference gene. The RefFinder got a comprehensive result that ranking the *eIF-4* as the most stable reference gene. In *Camellia sinensis*, the *eIF-4* was also selected as the best stable reference gene by the geNorm under the developmental stage^24^. And the phenomenon that different results were got using the different software was also found in *Pennisetum glaucum*^23^.

EPSPS is the target-set of glyphosate, from the shikimate pathway^43^. We determined the expression of *EPSPS* in the plants of a GR goosegrass population, caused by the amplification of the target set^16^. The expression of *EPSPS* shows a signification increase from 48 h to 72 h after treatment with glyphosate, as detected using the stable reference genes *ALS* and *eIF-4*. However, this phenomenon is not detected using the reference gene *TUB*. This result indicates that an appropriate reference is important for the precision of the result. Glufosinate is a popular non-selective herbicide with a target-site of GS and useful to control the GR weed species^15^. It is necessary to select the stable reference gene for the expression study due to the importance of this herbicide. In our research, the expression level of the reference genes *eIF-4* and *ALS* is similar to the *GS* in goosegrass. And these two reference genes show a higher stable than the *TUB* after glufosinate treated at different times. Several reports have identified ACCase inhibitor herbicide-resistant goosegrass worldwide. The expression level of *ACCase* is relatively similar to the reference gene 18S rRNA^9^. We also observed this phenomenon in our research, although the reference is different.

In summary, we found stables reference genes in goosegrass under the stress of three herbicides. Across different tissues and developmental stages, *eIF-4* is the best reference gene*. TUA* can serve as reference gene under glyphosate stress, whereas under glufosinate stress, *eIF-4* is the most stable reference gene. *UCE* can be used as reference gene under quizalofop-p-ethyl stress. Under stress from the three herbicides overall, *eIF-4* is the best reference gene for gene expression analysis.

## Methods

### Plant material and treatments

Seed samples of the goosegrass populations for this study were collected from the roadside of turf farms in Chengdu, Sichuan Province. The goosegrass seeds were cultured using the method in our previous research, with a single plant per pot^7^. When the plants reached the 4–5 leaves stage, tillering stage, and flowering fruit-bearing stage, the three types of herbicides were applied for stress treatment. The doses of these three herbicide were as follows: 840 g ae ha^−1^ of glyphosate (Roundup, isopropylamine salt of glyphosate, 410 g ae L^−1^, Monsanto Company, St. Louis, USA), 200 g a.i. ha^−1^ of Glufosinate (Basta^®^, 200 g L^−1^ glufosinate ammonium, soluble concentration (SL), Bayer CropScience, Frankfurt, Germany), and 37.5 g a.i. ha^−1^ of quizalofop-p-ethyl (50 g L^−1^ EC, Nissan chemical industry, Tokyo, Japan). The samples were collected and described as summarized in Table 3. For the tissues study, three organ types were harvested from the flowering fruit bearing stage. The fully emerged leaves were collected from three different developmental stages for the study of developmental stage. In the time treatment, the leaves were collected at the 4–5 leaf stage. Three biological replicates were performed and all samples were immediately frozen in liquid nitrogen and stored at −80 °C prior to RNA extraction.

### Total RNA isolation and cDNA synthesis

The frozen tissue was ground in liquid nitrogen using a mortar and pestle. Total RNA was extracted using the RNAprep Pure Plan Kit (Tiangen Biotech Beijing CO., LTD, Beijing, China). Genomic DNA was completely eliminated using RNase-Free DNase I (Tiangen Biotech Beijing CO., LTD, Beijing, China), according to the manufacturer’s instructions. The integrity was checked by 1.0% agarose gel electrophoresis. RNA and genomic DNA concentrations were determined using the NanoDrop-1000 spectrophotometer (NanoDrop Technologies, Wilmington, DE, USA). First-strand complementary DNA was synthesized using the FastQuant RT kit (Tiangen Biotech Beijing CO., LTD, Beijing, China) with 1 μg of total RNA and oligo (dT) in a total volume of 20 μL. The samples were stored at −80 °C until used.

### Candidate reference gene selection, primer design and gene cloning

9 common reference genes, *ACTIN, GAPDH, ALS, EF, eIF-4, TUA, TUB, UCE* and *HSP*, were selected for this study. The genes *TUA* and *TUB* were retrieved from the database of the National Centre for Biotechnology (NCBI). The other reference genes were obtained by querying the *E. indica* transcriptome database (SRR3614245) in our previous study. To confirm the sequences of the 9 candidate reference genes, all of them were cloned using polymerase chain reaction (PCR) and sequenced using the method described in our previous study^16^.

### qPCR assay

Primer pair sets for the confirmed candidate reference genes were designed using the software Oligo 7.0 (Table 1). The qPCR was performed in an ABI 7500 PCR machine utilizing SYBR Green detection chemistry (Applied Biosystems, Foster City, USA). The reaction mixture (25 μL) contained 12.5 μL 2 × Power SYBR Green PCR Master Mix, 1 μL of cDNA, 1 μL of each primer and 9.5 μL of ddH_2_O. The qPCR cycling conditions were as follows: 10 min at 95 °C, 40 cycles of 95 °C for 20 s and 60 °C for 1 min, then increasing the temperature by 0.5 °C every 5 s to obtain the product melt curve. All the qPCR assays included three technical and biological replicates. Standard curves were drawn to determine the amplification efficiency (E) and correlation coefficient (R^2^) of the diluted series based on the diluted (2×) cDNA series.

### Data analysis for expression stability

The software programs geNorm^32^, NormFinder^44^ and Bestkeeper^45^ were selected to calculate the expression stability of the 9 candidate reference genes. The web tool ReFinder^46^ was then selected to generate a comprehensive ranking for the reference genes by comparing the results calculated by these three software programs. The Cq values of all reference genes used in geNorm and NormFinder were converted into relative quantities according to the formula2^−∆Ct^ (∆Ct = the corresponding Cq value − minimum Cq)^47^. The geNorm program calculated the expression stability value (M), and reference genes with M < 1.5 were considered stable. The geNorm was also used to calculate the pairwise variation (V_n_/V_n+1_) between the two sequential normalization factors (NF_n_ and NF_n+1_) in order to determine the minimum number of reference genes for optimal normalization and the recommended cut-off value was 0.15^48^. NormFinder calculated the expression stabilities of the candidate reference genes by combining the intra- and inter-group variations in a sample set containing any number of samples organized in any number of groups. Raw Cq values were directly analysed by BestKeeper.

### The expression characteristics of *EPSPS, GS* and *ACCase* in goosegrass

To validate the stability of the selected reference genes for qPCR analysis under different herbicide stress, the expression of the target-site genes of glyphosate (*EPSPS*), glufosinate (*GS*) and quizalofop-p-ethyl (*ACCase*) were determined at different times (48 h, 72 h and 96 h) for the leaf tissues (4–5 leaves stage) after treatment. The plant culture and the herbicide treatment were performed using the methods described above. Both the most stable reference genes (*eIF-4, ALS*) and the most varying reference gene (*TUB*) were chosen for this study. The sequences of these three genes were retrieved from the transcript data in our previous study and re-confirmed^49^. The primer designing and qPCR methods were similar to the ones described above. *EPSPS, GS* and *ACCase* were amplified using the primer pairs (EP-F: 5′ GGTGGCAAGGTTAAGTTATCTGG 3′, EP-R: 5′ TCAACATAAGGGATGGAGATCAG 3′), (GS-F: 5′ GCGTGAAGATGGTGGATATGAAG 3′, GS-R: 5′ TTCGTGTTTCCCTGTCAACCTC 3′) and (ACC-F: 5′ TCATCATTCACATCAAACAGCATTCTC 3′, ACC-R: 5′ TGCCCATCACAATCCAACCAAAG 3′), respectively. The amount of transcript accumulated for these three genes normalized to the reference genes were analyzed using 2^−∆∆Ct^ method^47^. Three technical and biological replicates were performed in this study, and the data were statistically compared by one-way ANOVA.

## Additional Information

**How to cite this article:** Chen, J. *et al*. Selection of relatively exact reference genes for gene expression studies in goosegrass (*Eleusine indica*) under herbicide stress. *Sci. Rep.* **7**, 46494; doi: 10.1038/srep46494 (2017).

**Publisher's note:** Springer Nature remains neutral with regard to jurisdictional claims in published maps and institutional affiliations.

## Supplementary Material

Supplementary Information

## Figures and Tables

**Table 1:** The primer sequences and amplified characteristics of the 9 candidate reference genes for *E. indica.*

Gene symbol	Gene name	GeneBank Acession Number	Primer sequence (5′-3′) forward/reverse	Tm (°C)	Amplicon length (bp)	Efficiency (%)	Correlation coefficient
*ACTIN*	actin	KU720631	TCCCTGGTATCGCTGACCGTA	60	162	90.12	0.996
			CCCTTTGAGATCCACATCTGTT				
*GAPDH*	Glyceraldehyde-3-phosphate dehydrogenase	KU720632	TGCTATCACCGCTACCCAAA	60	98	94.92	0.991
			CAGTGCTGCTAGGAATGATG				
*ALS*	Chloroplast acetolactate synthase	KU720629	GCAATTTCCCCAGTGACGACC	60	152	93.06	0.990
			GCAAAAGCCTCTATCTTCCCTGT				
*EF*	Elongation factor 1-alpha	KU720633	GCTTCCAACTCCAAAGATGACCC	60	146	90.42	0.998
			TCAGCAAACTTCACAGCGAT				
*eIF-4*	Eukaryotic initiation factor 4 A	KU720634	CCTACCAAAACGACCACTACGAC	60	126	90.78	0.998
			ATCACCACCGACCTCCTTGCTC				
*TUA*	Alpha tubulin 1	AF008120.1	CCCCGTGCCGTGTTTGTT	60	108	97.25	0.997
			ATCCTCCTTGCCACTGATGAGC				
*TUB*	Beta-tubulin 4	AF059290.1	GGACGAGATGGAGTTCACCGAG	60	117	94.89	0.995
			GCCTCATCCTCATCCTCGTAGT				
*UCE*	Ubiquitin-conjugating enzyme	KX817291.1	TGCCCCACACAAGAGAATATGCC	60	131	92.81	0.998
			TTCCAGTTGTCAGAGCATCATCC				
*HSP*	Heat shock protein 70	KU720635.1	GACTCACAAAGGACAGCAACAAAA	60	196	90.82	0.994
			CCTCAAAAACACCATCACCGACTTCA				

**Table 2:** Gene expression stability ranked by geNorm, NormFinder, BestKeeper and RefFinder.

Group	Rank	GeNorm		NormFinder		Bestkeeper			RefFinder	
		Gene	Stability	Gene	Stability	Gene	SD[ ± Cq]	CV (%Cq)	Gene	Stability
Control	1	*eIF-4*	0.54	*eIF-4*	0.24	*TUA*	0.88	3.93	*eIF-4*	1.41
	2	*EF*	0.54	*ALS*	0.35	*ACTIN*	0.93	3.66	*UCE*	3.00
	3	*UCE*	0.73	*UCE*	0.46	*UCE*	0.95	4.00	*ALS*	3.13
	4	*ALS*	0.79	*EF*	0.55	*eIF-4*	1.00	4.46	*EF*	3.36
	5	*GAPDH*	1.12	*GAPDH*	0.93	*TUB*	1.03	4.23	*TUA*	4.30
	6	*ACTIN*	1.34	*ACTIN*	1.02	*ALS*	1.13	4.58	*ACTIN*	4.56
	7	*TUA*	1.48	*TUA*	1.14	*GAPDH*	1.32	4.36	*GAPDH*	5.44
	8	*TUB*	1.60	*TUB*	1.23	*EF*	1.36	6.42	*TUB*	7.11
	9	*HSP*	1.72	*HSP*	1.33	*HSP*	2.10	8.64	*HSP*	9.00
Glyphosate	1	*eIF-4*	0.58	*ALS*	0.71	*HSP*	0.74	3.04	*ALS*	1.50
	2	*ALS*	0.58	*TUA*	0.72	*ACTIN*	0.81	3.12	*TUA*	2.63
	3	*EF*	0.86	*EF*	0.74	*TUA*	0.81	3.71	*eIF-4*	3.13
	4	*TUA*	1.20	*eIF-4*	0.85	*GAPDH*	1.31	4.60	*EF*	3.71
	5	*GAPDH*	1.48	*GAPDH*	0.90	*ALS*	1.37	5.63	*HSP*	3.83
	6	*HSP*	1.63	*HSP*	0.99	*eIF-4*	1.71	7.84	*GAPDH*	4.73
	7	*ACTIN*	1.68	*ACTIN*	1.07	*EF*	1.96	9.55	*ACTIN*	5.12
	8	*UCE*	1.85	*UCE*	1.43	*UCE*	1.97	8.93	*UCE*	8.00
	9	*TUB*	2.06	*TUB*	1.79	*TUB*	2.80	11.22	*TUB*	9.00
Glufosinate	1	*eIF-4*	0.67	*eIF-4*	0.47	*UCE*	0.47	1.95	*eIF-4*	1.63
	2	*ALS*	0.67	*ALS*	0.55	*ACTIN*	0.67	2.72	*ALS*	2.34
	3	*TUA*	0.93	*TUA*	0.59	*TUA*	0.96	4.32	*UCE*	2.38
	4	*UCE*	1.22	*UCE*	0.63	*GAPDH*	1.10	3.75	*TUA*	3.22
	5	*ACTIN*	1.38	*EF*	0.90	*ALS*	1.23	5.00	*ACTIN*	5.42
	6	*GAPDH*	1.47	*GAPDH*	0.98	*HSP*	1.25	5.05	*GAPDH*	5.42
	7	*HSP*	1.53	*ACTIN*	1.02	*eIF-4*	1.39	6.31	*EF*	6.88
	8	*EF*	1.64	*HSP*	2.17	*EF*	1.66	7.89	*HSP*	7.20
	9	*TUB*	2.00	*TUB*	2.03	*TUB*	2.69	10.96	*TUB*	9.00
Quizalofop-p-ethyl	1	*eIF-4*	0.47	*eIF-4*	0.16	*TUA*	0.48	2.15	*UCE*	1.32
	2	*UCE*	0.47	*UCE*	0.16	*EF*	0.53	2.48	*eIF-4*	2.11
	3	*EF*	0.59	*EF*	0.31	*UCE*	0.71	2.99	*EF*	2.71
	4	*ACTIN*	0.69	*ACTIN*	0.49	*ACTIN*	0.88	3.47	*TUA*	3.66
	5	*ALS*	0.79	*TUA*	0.57	*eIF-4*	0.89	3.91	*ACTIN*	4.00
	6	*TUA*	0.87	*ALS*	0.59	*GAPDH*	1.17	4.16	*ALS*	5.69
	7	*GAPDH*	0.97	*GAPDH*	0.68	*ALS*	1.21	4.75	*GAPDH*	6.74
	8	*HSP*	1.12	*HSP*	1.11	*TUB*	1.31	5.07	*HSP*	8.24
	9	*TUB*	1.34	*TUB*	1.39	*HSP*	1.77	7.25	*TUB*	8.74
Total	1	*eIF-4*	0.90	*eIF-4*	0.48	*TUA*	0.84	3.77	*eIF-4*	1.50
	2	*ALS*	0.90	*ALS*	0.67	*ACTIN*	0.93	3.67	*ALS*	2.00
	3	*EF*	1.18	*EF*	0.78	*UCE*	1.17	4.99	*TUA*	2.83
	4	*GAPDH*	1.48	*GAPDH*	0.90	*ALS*	1.25	5.06	*EF*	3.71
	5	*TUA*	1.62	*ACTIN*	0.91	*eIF-4*	1.28	5.75	*UCE*	4.40
	6	*ACTIN*	1.69	*TUA*	0.99	*GAPDH*	1.31	4.55	*ACTIN*	4.56
	7	*UCE*	1.74	*UCE*	1.01	*EF*	1.38	6.56	*GAPDH*	6.96
	8	*HSP*	1.82	*HSP*	1.30	*HSP*	1.41	5.76	*HSP*	7.74
	9	*TUB*	2.09	*TUB*	1.94	*TUB*	2.06	8.26	*TUB*	9.00

SD[± Cq]: standard deviation of the Cq; CV[%Cq]: coefficient of variance expressed as a percentage of the Cq level.

**Table 3:** The summary of the samples harvested at different growth stage and herbicide treatment in this study.

Stress treatment	Tissues^a^			Growing stage^b^			Times after herbicide treatment		
	Leaves^d^	Stem^e^	Panicle	4–5 leaves	Tillering stage	Booting stage	24 H	48 H	72 H
Control	√	√	√	√	√	√	×	×	×
Glyphosate	√	√	√	√	√	√	√	√	√
Glufosinate	√	√	√	√	√	√	√	√	√
Quizalofop-p-ethyl	√	√	√	√	√	√	√	√	√

^a^The different tissues were collected at the stage of flowering fruit bearing stage.

^b^The leaves of three uppermost fully expanded were selected in the growing stage group.

^c^The organ of leaves were selected in the study of different time under the stress of three kinds of herbicide.

^d^Three uppermost fully expanded leaves were selected as samples.

^e^Approximately 4–5 cm of stem selected from one of the tillers.

## References

[b1] Oerke E.-C. Crop losses to pests. The Journal of Agricultural Science 144, 31–43 (2006).

[b2] Heap I. In Integrated Pest Management (eds Dayid Pimentel & Rajinder Peshin) 281–301 (Springer, 2014).

[b3] Heap I. The International Survey of Herbicide Resistant Weeds http://www.weedscience.org (2016).

[b4] Holm L. G., Plucknett D. L., Pancho J. V. & Herberger J. P. The world’s worst weeds: Distribution and Biology. p, (609) (University Press of Hawaii, Hawaii, 1977).

[b5] Corbett J. L., Askew S. D., Thomas W. E. & Wilcut J. W. Weed Efficacy Evaluations for Bromoxynil, Glufosinate, Glyphosate, Pyrithiobac, and Sulfosate1. Weed Technology 18, 443–453 (2004).

[b6] Lee L. J. & Ngim J. A first report of glyphosate‐resistant goosegrass (*Eleusine indica* (L) Gaertn) in Malaysia. Pest Management Science 56, 336–339 (2000).

[b7] Chen J., Huang H., Wei S., Zhang C. & Huang Z. Characterization of glyphosate-resistant goosegrass (*Eleusine indica*) populations in China. Journal of Integrative Agriculture 14, 919–925 (2015).

[b8] McCullough P. E., Yu J., Raymer P. & Chen Z. First Report of ACCase-Resistant Goosegrass (*Eleusine indica*) in the United States. Weed Science 64, 399–408 (2016).

[b9] San Cha T., Najihah M. G., Sahid I. B. & Chuah T. S. Molecular basis for resistance to ACCase-inhibiting fluazifop in *Eleusine indica* from Malaysia. Pesticide Biochemistry and Physiology 111, 7–13 (2014). 24861927 10.1016/j.pestbp.2014.04.011

[b10] Jalaludin A., Ngim J., Bakar B. H. & Alias Z. Preliminary findings of potentially resistant goosegrass (*Eleusine indica*) to glufosinate‐ammonium in Malaysia. Weed Biology and Management 10, 256–260 (2010).

[b11] Jalaludin A., Yu Q. & Powles S. Multiple resistance across glufosinate, glyphosate, paraquat and ACCase‐inhibiting herbicides in an *Eleusine indica* population. Weed Research 55, 82–89 (2015).

[b12] Seng C. T., Van Lun L., San C. T. & SAHID I. B. Initial report of glufosinate and paraquat multiple resistance that evolved in a biotype of goosegrass (*Eleusine indica*) in Malaysia. Weed Biology and Management 10, 229–233 (2010).

[b13] Délye C., Jasieniuk M. & Le Corre V. Deciphering the evolution of herbicide resistance in weeds. Trends in Genetics 29, 649–658 (2013). 23830583 10.1016/j.tig.2013.06.001

[b14] Powles S. B. & Yu Q. Evolution in action: plants resistant to herbicides. Annual Review of Plant Biology 61, 317–347 (2010). 10.1146/annurev-arplant-042809-112119 20192743

[b15] Gaines T. A. . RNA-Seq transcriptome analysis to identify genes involved in metabolism-based diclofop resistance in *Lolium rigidum*. The Plant Journal 78, 865–876 (2014). 24654891 10.1111/tpj.12514

[b16] Chen J. . Mutations and amplification of *EPSPS* gene confer resistance to glyphosate in goosegrass (*Eleusine indica*). Planta 242, 1–10 (2015). 25998526 10.1007/s00425-015-2324-2

[b17] Cummins I. . Key role for a glutathione transferase in multiple-herbicide resistance in grass weeds. Proceedings of the National Academy of Sciences 110, 5812–5817 (2013). 10.1073/pnas.1221179110 PMC3625300 23530204

[b18] Derveaux S., Vandesompele J. & Hellemans J. How to do successful gene expression analysis using real-time PCR. Methods 50, 227–230 (2010). 19969088 10.1016/j.ymeth.2009.11.001

[b19] Bustin S. A. . The MIQE guidelines: minimum information for publication of quantitative real-time PCR experiments. Clinical Chemistry 55, 611–622 (2009). 19246619 10.1373/clinchem.2008.112797

[b20] Bustin S. A. . MIQE precis: Practical implementation of minimum standard guidelines for fluorescence-based quantitative real-time PCR experiments. BMC Molecular Biology 11, 74, 10.1186/1471-2199-11-74 (2010). 20858237 PMC2955025

[b21] Martins P. K. . Selection of reliable reference genes for RT-qPCR analysis during developmental stages and abiotic stress in *Setaria viridis*. Scientific Reports 6, 28348, 10.1038/srep28348 (2016). 27321675 PMC4913262

[b22] Zhang S., Zeng Y., Yi X. & Zhang Y. Selection of suitable reference genes for quantitative RT-PCR normalization in the halophyte *Halostachys caspica* under salt and drought stress. Scientific Reports 6, 30363, 10.1038/srep30363 (2016). 27527518 PMC4985824

[b23] Shivhare R. & Lata C. Selection of suitable reference genes for assessing gene expression in pearl millet under different abiotic stresses and their combinations. Scientific Reports 6, 23036; 10.1038/srep23036 (2016). 26972345 PMC4789795

[b24] Wu Z. J., Tian C., Jiang Q., Li X. H. & Zhuang J. Selection of suitable reference genes for qRT-PCR normalization during leaf development and hormonal stimuli in tea plant (*Camellia sinensis*). Scientific Reports 6, 19748; 10.1038/srep19748 (2016). 26813576 PMC4728435

[b25] Xu X. . Selection of relatively exact reference genes for gene expression studies in flixweed (*Descurainia sophia*) by quantitative real-time polymerase chain reaction. Pesticide Biochemistry and Physiology 127, 59–66 (2016). 26821659 10.1016/j.pestbp.2015.09.007

[b26] Wrzesińska B., Kierzek R. & Obrępalska‐Stęplowska A. Evaluation of six commonly used reference genes for gene expression studies in herbicide‐resistant *Avena fatua* biotypes. Weed Research 56, 284–292 (2016).

[b27] Petit C., Pernin F., Heydel J. M. & Délye C. Validation of a set of reference genes to study response to herbicide stress in grasses. BMC Research Notes 5, 18; 10.1186/1756-0500-5-18 (2012). 22233533 PMC3292489

[b28] Duhoux A. & Délye C. Reference genes to study herbicide stress response in *Lolium sp*.: up-regulation of P450 genes in plants resistant to acetolactate-synthase inhibitors. PLoS One 8, e63576; 10.1371/journal.pone.0063576 (2013). 23696834 PMC3656029

[b29] ZHANG C. . Investigating the mechanisms of glyphosate resistance in goosegrass (*Eleusine indica*) population from South China. Journal of Integrative Agriculture 14, 909–918 (2015).

[b30] Huffman J. L., Riggins C. W., Steckel L. E. & Tranel P. J. The EPSPS Pro106Ser substitution solely accounts for glyphosate resistance in a goosegrass (*Eleusine indica*) population from Tennessee, United States. Journal of Integrative Agriculture 15, 1304–1312 (2016).

[b31] Gomes M. P. . Alteration of plant physiology by glyphosate and its by-product aminomethylphosphonic acid: an overview. Journal of Experimental Botany 65, 4691–4703 (2014). 25039071 10.1093/jxb/eru269

[b32] Vandesompele J. . Accurate normalization of real-time quantitative RT-PCR data by geometric averaging of multiple internal control genes. Genome Biology 3, 1; 10.1186/gb-2002-3-7-research0034 (2002). PMC126239 12184808

[b33] Gaines T. A. . Gene amplification confers glyphosate resistance in *Amaranthus palmeri*. Proceedings of the National Academy of Sciences 107, 1029–1034 (2010). 10.1073/pnas.0906649107 PMC2824275 20018685

[b34] Salas R. A., Scott R. C., Dayan F. E. & Burgos N. R. *EPSPS* Gene Amplification in Glyphosate-Resistant Italian Ryegrass (*Lolium perenne* ssp. *multiflorum*) Populations from Arkansas, USA. Journal of Agricultural and Food Chemistry 63, 5885–5893 (2015). 25760654 10.1021/acs.jafc.5b00018

[b35] Peng Y. . Characterization of the horseweed (*Conyza canadensis*) transcriptome using GS-FLX 454 pyrosequencing and its application for expression analysis of candidate non-target herbicide resistance genes. Pest Management Science 66, 1053–1062 (2010). 20715018 10.1002/ps.2004

[b36] Riggins C. W., Peng, Y. Jr, C. N. S. & Tranel P. J. Characterization of de novo transcriptome for waterhemp (*Amaranthus tuberculatus*) using GS-FLX 454 pyrosequencing and its application for studies of herbicide target-site genes. Pest Management Science 66, 1042–1052 (2010). 20680963 10.1002/ps.2006

[b37] Dombrowski J. E. & Martin R. C. Evaluation of reference genes for quantitative RT-PCR in *Lolium temulentum* under abiotic stress. Plant Science 176, 390–396 (2008).

[b38] Jain M., Nijhawan A., Tyagi A. K. & Khurana J. P. Validation of housekeeping genes as internal control for studying gene expression in rice by quantitative real-time PCR. Biochemical and Biophysical Research Communications 345, 646–651 (2006). 16690022 10.1016/j.bbrc.2006.04.140

[b39] Hong S. Y., Seo P. J., Yang M. S., Xiang F. & Park C. M. Exploring valid reference genes for gene expression studies in *Brachypodium distachyon* by real-time PCR. BMC Plant Biology 8, 112; 10.1186/1471-2229-8-112 (2008). 18992143 PMC2588586

[b40] Faccioli P. . A combined strategy of “*in silico*” transcriptome analysis and web search engine optimization allows an agile identification of reference genes suitable for normalization in gene expression studies. Plant Molecular Biology 63, 679–688 (2007). 17143578 10.1007/s11103-006-9116-9

[b41] Malone J. M., Morran S., Shirley N., Boutsalis P. & Preston C. *EPSPS* gene amplification in glyphosate-resistant *Bromus diandrus*. Pest Management Science 72, 81–88 (2015). 25847720 10.1002/ps.4019

[b42] Wiersma A. T. . Gene amplification of 5-enol-pyruvylshikimate-3-phosphate synthase in glyphosate-resistant *Kochia scoparia*. Planta, 1–12 (2014). 10.1007/s00425-014-2197-9 25366557

[b43] Duke S. O. & Powles S. B. Glyphosate: a once-in-a-century herbicide. Pest Management Science 64, 319–325 (2008). 18273882 10.1002/ps.1518

[b44] Andersen C. L., Jensen J. L. & Ørntoft T. F. Normalization of real-time quantitative reverse transcription-PCR data: a model-based variance estimation approach to identify genes suited for normalization, applied to bladder and colon cancer data sets. Cancer Research 64, 5245–5250 (2004). 15289330 10.1158/0008-5472.CAN-04-0496

[b45] Pfaffl M. W., Tichopad A., Prgomet C. & Neuvians T. P. Determination of stable housekeeping genes, differentially regulated target genes and sample integrity: BestKeeper–Excel-based tool using pair-wise correlations. Biotechnology Letters 26, 509–515 (2004). 15127793 10.1023/b:bile.0000019559.84305.47

[b46] Xie F., Xiao P., Chen D., Xu L. & Zhang B. miRDeepFinder: a miRNA analysis tool for deep sequencing of plant small RNAs. Plant Molecular Biology 80, 75–84 (2012). 10.1007/s11103-012-9885-2 22290409

[b47] Livak K. J. & Schmittgen T. D. Analysis of Relative Gene Expression Data Using Real-Time Quantitative PCR and the 2^−ΔΔCT^ Method. Methods 25, 402–408 (2001). 11846609 10.1006/meth.2001.1262

[b48] Hellemans J., Mortier G., De Paepe A., Speleman F. & Vandesompele J. qBase relative quantification framework and software for management and automated analysis of real-time quantitative PCR data. Genome Biology 8, R19; 10.1186/gb-2007-8-2-r19 (2007). 17291332 PMC1852402

[b49] Chen J. . Investigating the mechanisms of glyphosate resistance in goosegrass (*Eleusine indica* (L.) Gaertn.) by RNA Sequencing technology. The Plant Journal 89, 407–415 (2017) 27743420 10.1111/tpj.13395

