# Supplementary material for: Selection of relatively exact reference genes for gene expression studies in goosegrass (Eleusine indica) under herbicide stress
Source: Sci Rep. 2017 Apr 21;7:46494. doi: 10.1038/srep46494 (PMC5399354; doi:10.1038/srep46494)
Supplement: Supplementary Information [file srep46494-s1.pdf]

Selection of relatively exact reference genes for gene expression studies in goosegrass (*Eleusine indica*) under herbicide stress

Jingchao Chen[1], Zhaofeng Huang[1], Hongjuan Huang[1], Shouhui Wei[1], Yan Liu[2], Cuilan Jiang[1], Jie Zhang[3], Chaoxian Zhang[1,*]

1. Key Laboratory of Weed and Rodent Biology and Management, Institute of Plant Protection, Chinese Academy of Agricultural Sciences, Beijing 100193, P. R. China.
2. Environment and Plant Protection Institute, Chinese Academy of Tropical Agricultural Sciences, Danzhou 571737, P. R. China.
3. State Key Laboratory for Biology of Plant Diseases and Insect Pests, Institute of Plant Protection, Chinese Academy of Agricultural Sciences, Beijing 100193, P. R. China.

*Corresponding author: Chaoxian Zhang, Professor, Tel: +86-010-62815937, Fax: +86-010-62810289, E-mail: cxzhang@ippcaas.cn

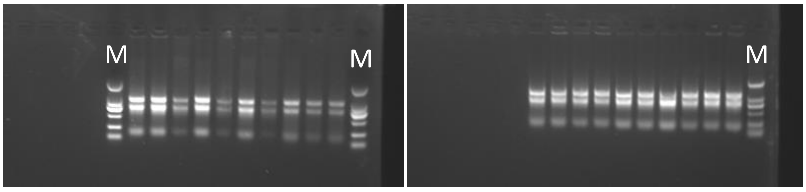

Supplementary Figure S1. The total RNA extraction results detected by the gel electrophoresis for parts of the samples. M: DL 2000 maker.

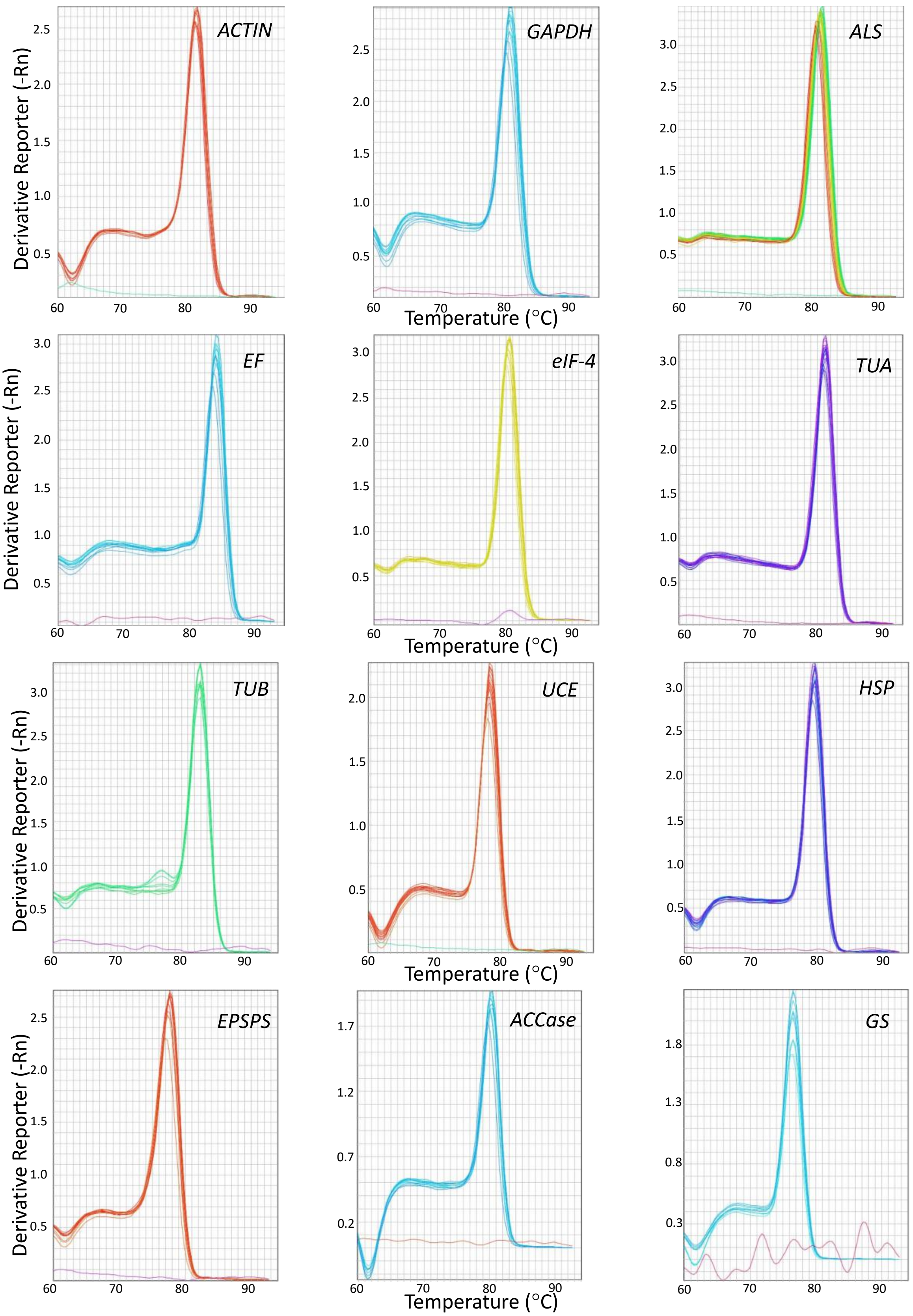

Supplementary Figure S2. Melt curve analyses of 9 candidate reference genes and three validated genes in *E. indica* for qPCR.

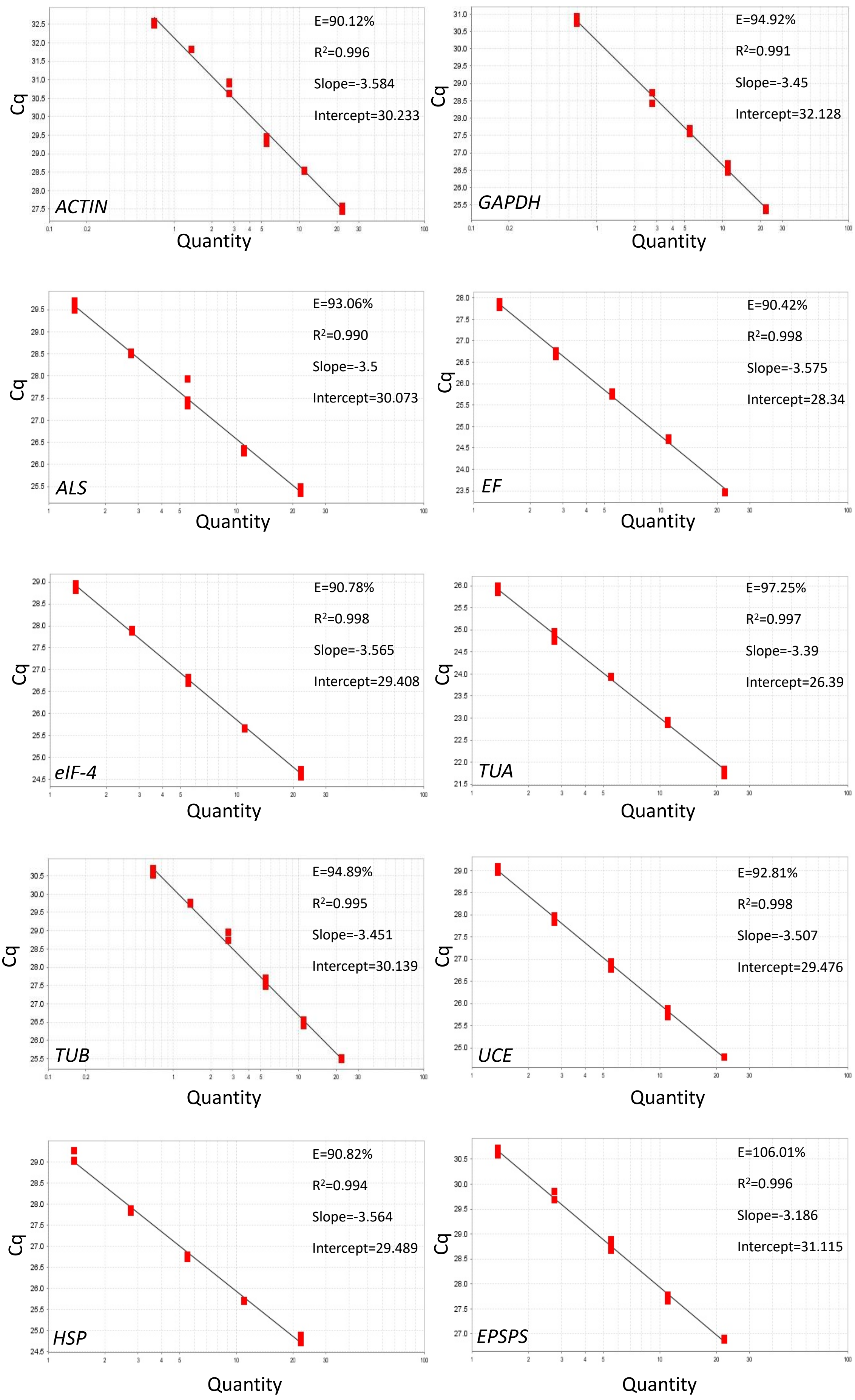
ACTIN
E=90.12%
R^2=0.996
Slope=-3.584
Intercept=30.233
Cq
Quantity
GAPDH
E=94.92%
R^2=0.991
Slope=-3.45
Intercept=32.128
Cq
Quantity
ALS
E=93.06%
R^2=0.990
Slope=-3.5
Intercept=30.073
Cq
Quantity
EF
E=90.42%
R^2=0.998
Slope=-3.575
Intercept=28.34
Cq
Quantity
eIF-4
E=90.78%
R^2=0.998
Slope=-3.565
Intercept=29.408
Cq
Quantity
TUA
E=97.25%
R^2=0.997
Slope=-3.39
Intercept=26.39
Cq
Quantity
TUB
E=94.89%
R^2=0.995
Slope=-3.451
Intercept=30.139
Cq
Quantity
UCE
E=92.81%
R^2=0.998
Slope=-3.507
Intercept=29.476
Cq
Quantity
HSP
E=90.82%
R^2=0.994
Slope=-3.564
Intercept=29.489
Cq
Quantity
EPSPS
E=106.01%
R^2=0.996
Slope=-3.186
Intercept=31.115
Cq
Quantity

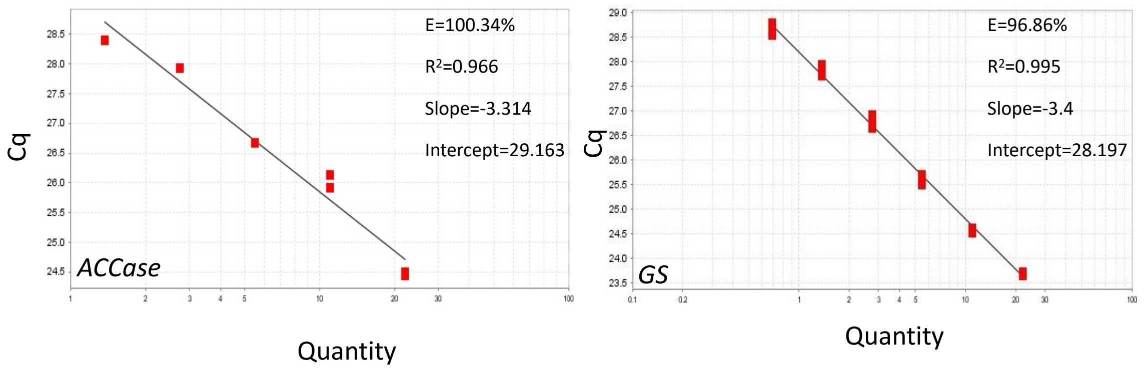

Supplementary Figure S3. Standard curve of 9 candidate reference genes and three validated genes in *E. indica* for qPCR

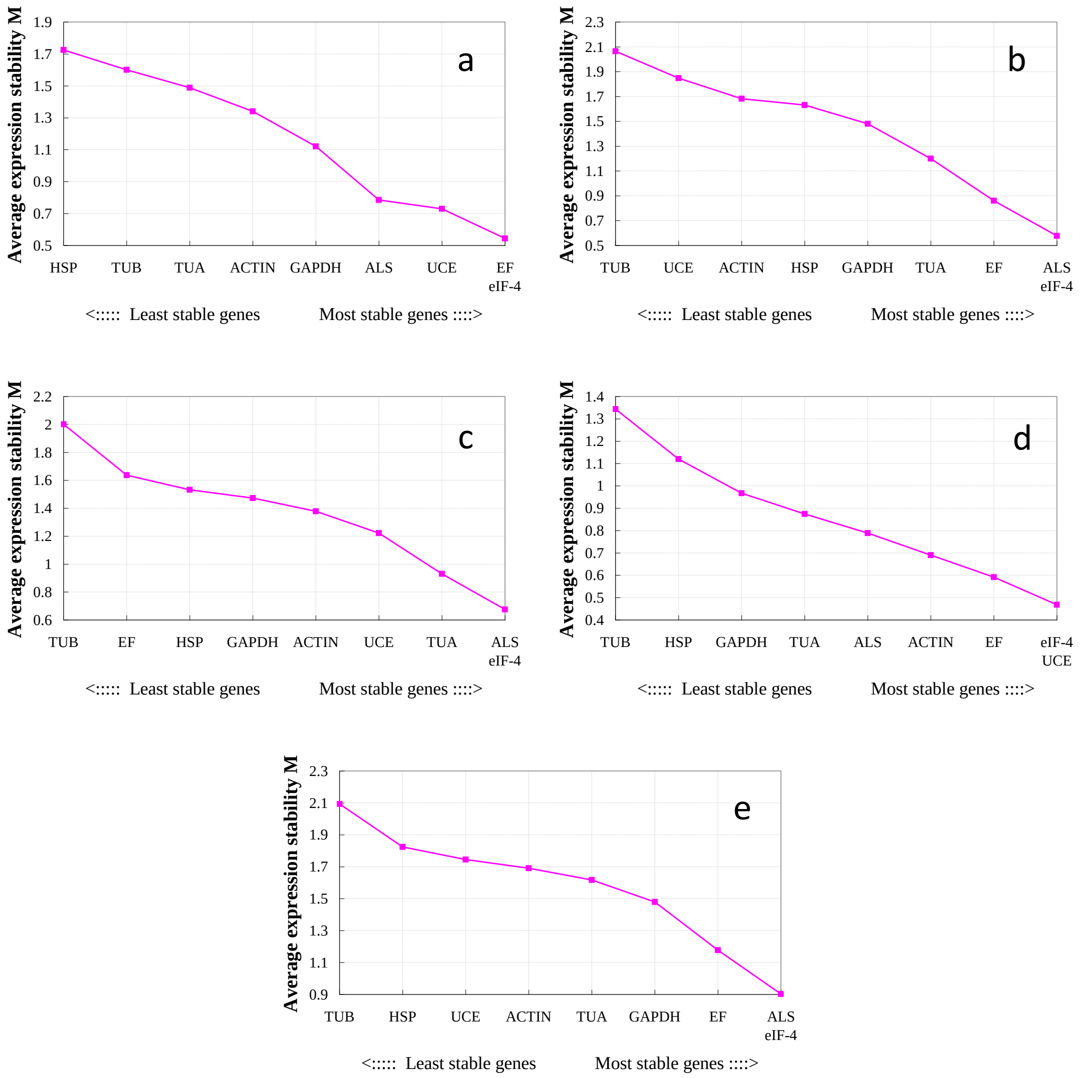

Supplementary Figure S4. Average expression stability values (M) calculated by geNorm. A lower value of average expression stability (M) indicates most stable expression. (a) Control; (b) Glyphosate; (c) Glufosinate; (d) Quizalofop-p-ethyl; (e) Total.

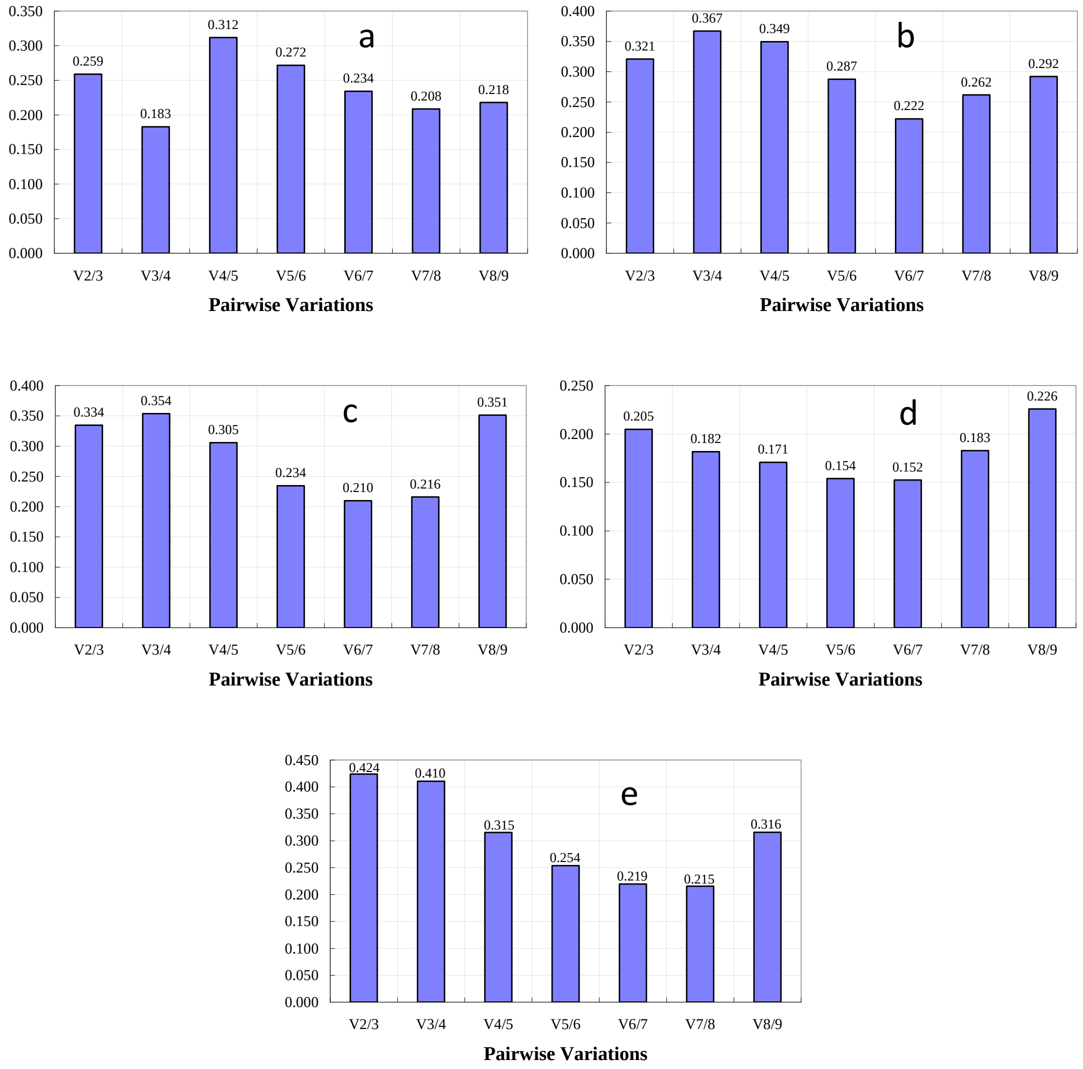

Supplementary Figure S5. Pairwise variation (V) to define the optimal number of reference gene required to a reliable normalization to each dataset. (a) Control; (b) Glyphosate; (c) Glufosinate; (d) Quizalofop-p-ethyl; (e) Total.
